# Neurophysiological contributors to advantageous risk-taking: an experimental psychopharmacological investigation

**DOI:** 10.1093/scan/nsab047

**Published:** 2021-04-16

**Authors:** Jennifer K MacCormack, Emma Armstrong-Carter, Kathryn L Humphreys, Keely A Muscatell

**Affiliations:** Department of Psychology and Neuroscience, University of North Carolina at Chapel Hill, Chapel Hill, NC 27599, USA; Department of Psychiatry, University of Pittsburgh, Pittsburgh, PA 15260, USA; Graduate School of Education, Stanford University, Stanford, CA 94305, USA; Department of Psychology and Human Development, Vanderbilt University, Nashville, USA; Department of Psychology and Neuroscience, University of North Carolina at Chapel Hill, Chapel Hill, NC 27599, USA; Lineberger Comprehensive Cancer Center, University of North Carolina at Chapel Hill, Chapel Hill, NC 27599, USA; Carolina Population Center, University of North Carolina at Chapel Hill, Chapel Hill, NC 27599, USA

**Keywords:** beta-adrenergic blockade, propranolol, learning, risk-taking, arousal

## Abstract

The ability to learn from experience is critical for determining when to take risks and when to play it safe. However, we know little about how within-person state changes, such as an individual’s degree of neurophysiological arousal, may impact the ability to learn which risks are most likely to fail *vs* succeed. To test this, we used a randomized, double-blind, placebo-controlled design to pharmacologically manipulate neurophysiological arousal and assess its causal impact on risk-related learning and performance. Eighty-seven adults (45% female, *M*_age_ = 20.1 ± 1.46 years) took either propranolol (*n* = 42), a beta-adrenergic receptor blocker that attenuates sympathetic nervous system–related signaling, or a placebo (*n* = 45). Participants then completed the Balloon Emotional Learning Task, a risk-taking task wherein experiential learning is necessary for task success. We found that individuals on propranolol, relative to placebo, earned fewer points on the task, suggesting that they were less effective risk-takers. This effect was mediated by the fact that those on propranolol made less optimal decisions in the final phase of the task on trials with the greatest opportunity for advantageous risk-taking. These findings highlight that neurophysiological arousal supports risk-related learning and, in turn, more advantageous decision-making and optimal behavior under conditions of risk.

Everyday life is filled with situations in which we must decide whether to take a risk or play it safe. Should we ask that attractive stranger for their number, try out that new restaurant, or risk not getting a health concern examined? Effective risky decision-making does not just involve estimations of chance but also requires learning from prior information and experience in order to predict the likelihood of positive or negative outcomes (Denrell, 2007; Ben-Elia *et al.*, 2008). One classic illustration of learning-informed risk-taking is how drivers learn over time which roads have the least risk of traffic given the time of the day, weather conditions, etc. For example, during rush hour, drivers might risk using a shortcut but discover that this risky choice proved worse than their typical route, reducing their likelihood to risk similar shortcuts in the future during comparable traffic conditions.

Ultimately, what factors contribute to people’s ability to learn from experience in order to optimize when to play it safe *vs* take a risk? Most prior literature investigates the role of trait-based factors such as impulsivity or sensation-seeking in predicting risk-taking (e.g. Nigg, 2017; Khurana *et al.*, 2018). This focus on trait-based predictors means we still know little about how state factors within the individual impact risk-taking, especially in contexts where experiential learning is critical to success. One long-standing state factor of interest has been arousal, with prior theory suggesting that some arousal is beneficial or facilitative for decision-making, especially when decisions are more intuitive, uncertain, ambiguous or risky (e.g. Bechara *et al.*, 2000; Storbeck and Clore, 2008). Furthermore, a certain degree of arousal can support effective learning, as arousal helps sustain the attention needed for noticing and encoding information while also potentially promoting the acquisition of feedback for learning via exploration and experimentation (e.g. Yerkes and Dodson, 1908; Aston-Jones and Cohen, 2005; Eldar *et al.*, 2013). Arousal can be operationalized in several ways—subjectively (e.g. self-report), behaviorally (e.g. pupil dilation) or neurophysiologically [e.g. sympathetic nervous system (SNS) signaling]. Herein, we manipulated SNS-related neurophysiological arousal using the beta-blocker drug propranolol and investigated subsequent effects on risk-taking behavior in a task that requires learning from experience, with the key prediction that propranolol would ultimately impair advantageous risk-taking.

## Advantageous risk-taking involves learning from experience

Building more accurate predictions from past experience (i.e. learning) is key for guiding advantageous risk decisions in real life outside the laboratory (Lo and Repin, 2002; Denrell, 2007; Ben-Elia *et al.*, 2008). However, most prior laboratory research examines risk-taking in the context of gambling-based chance games wherein there is little opportunity for learning (Lejuez *et al.*, 2002; Rogers *et al.*, 2004; FeldmanHall *et al.*, 2016). When learning-guided risk-taking is studied in the laboratory, the Iowa Gambling Task (Bechara *et al.,* 1994) is often used. In this task, participants choose cards from four decks with different—initially unknown—average reward and punishment contingencies. As an implicit learning task, participants must learn from successive trials which decks produce advantageous *vs* disadvantageous outcomes. Although the Iowa Gambling Task allows for a behavioral test following implicit learning, the choices are forced choice (i.e. participants must choose a card) and categorical, resulting in fewer opportunities to learn and explore within each trial.

To address the need for a more dimensional, learning-driven risk-taking task, Humphreys and colleagues (Humphreys *et al.*, 2013) created the Balloon Emotional Learning Task (BELT). In the BELT, individuals have more opportunity within each trial to explore the bounds of risk-taking (e.g. via balloon pumps) while learning across multiple trials which conditions afford more advantageous *vs* disadvantageous risks. The BELT thus offers an improvement over other implicit learning tasks (e.g. Iowa Gambling Task), as it captures more dimensional decision-based processes in contexts that support greater exploration within each trial rather than forced-choice decisions. Initial studies using the BELT suggested that a combination of dispositional factors is associated with maximal task performance (Humphreys *et al.*, 2013). However, less research has examined the intraindividual mechanisms that contribute to learning about advantageous risk-taking. As such, we know little about how within-person state fluctuations influence learning about when it is most effective to take risks.

## Theoretical role of arousal in risk-taking

Both theory and empirical research identify arousal as one fundamental intraindividual pathway that facilitates learning and effective risk-taking. Arousal supports diverse functions such as wakefulness, motivational states, attention to salient or evocative stimuli, encoding and retrieval in learning and memory, and affect-based perceptions and decisions (see discussion in Satpute *et al.*, 2019). Arousal is derived from the integration of afferent autonomic signals from the periphery (e.g. the SNS) alongside signals from other neuromodulating pathways such as the adrenergic/noradrenergic, serotoninergic and dopaminergic systems (e.g. Robbins and Everitt, 1995; Coull *et al.*, 1997; Critchley *et al.*, 2000; Berridge, 2008; Kleckner *et al.*, 2017; Satpute *et al.*, 2019).

More generally, theories of arousal such as affect-as-information theory posit that individuals implicitly use their momentary feelings (e.g. arousal rooted in afferent physiological signals) to evaluate contextual cues and make decisions that drive behavior (Schachter and Singer, 1962; Clore *et al.*, 2001; Storbeck and Clore, 2008; Schwarz, 2010). Similarly, the somatic marker hypothesis suggests that physiological sensations during and after decisions help individuals better determine whether it will be advantageous to make that decision again in future (Damasio, 1994, 1999; Bechara *et al.*, 1999). Finally, predictive inference models of affect argue that the brain uses both a priori knowledge and ongoing afferent physiological signals (including arousal) to interpret contextual cues and inform behavior (Barrett and Simmons, 2015; Barrett, 2017; MacCormack and Lindquist, 2017). Ultimately, these theories suggest that the predictions built through experiential learning should interact with the neurobiology underpinning arousal to improve decision-making under conditions of uncertainty (e.g. risk). Risky decisions, as assessed in this study with a learning task rather than a gambling task and combined with the power of pharmacological blockade, provide a valuable model for testing these theory-driven hypotheses.

## Neurobiological evidence for arousal in learning and risk-taking

Consistent with the theoretical insights above, a long history of studies in animals and humans show that arousal and arousal-related neurobiology are key mediators of effective learning and risk-taking. For instance, economic traders who exhibited greater autonomic responses during market trades and those who were more interoceptively aware of their physiological sensations make more advantageous decisions compared with their colleagues (Lo and Repin, 2002; Kandasamy *et al.*, 2016). Arousal also appears to influence gamblers’ ability to judge situations and risks effectively (Tranel, 2000). Conversely, blunted arousal or the impaired ability to perceive physiological signaling may hinder learning about advantageous risk-taking (Critchley *et al.*, 2001). For example, during the Iowa Gambling Task, individuals with medical conditions that weaken afferent peripheral signals selected riskier options and performed worse compared with healthy individuals (Busemeyer and Stout, 2002; Yechiam *et al.*, 2005). Neurophysiological arousal may be particularly important for guiding decisions in ambiguous contexts when more information is needed to perform optimally (Zink *et al.*, 2004; FeldmanHall *et al.*, 2016).

Not only is there promising behavioral evidence for the role of arousal in facilitating advantageous risk-taking, but also there is compelling neurobiological evidence that the SNS and adrenergic/noradrenergic systems matter for both learning and risk-taking (Sara, 2009). The SNS is a fast-acting branch of the autonomic nervous system that helps initiate changes across the cardiovascular system and other modalities (e.g. pupil dilation, sweat) in response to environmental stimuli. As such, the SNS facilitates heightened action-readiness and vigilance to environmental cues, providing richer information when making decisions (Ruffolo, 1991; Blascovich *et al.*, 2011). SNS activation itself is largely instigated by the adrenergic/noradrenergic systems via binding of the catecholamines epinephrine and norepinephrine to beta-adrenergic receptors throughout the body and brain.

Classic rodent experiments demonstrate that knockout, lesioning or blockade of SNS-related neurobiology reduces learning across multiple domains (e.g. motor, spatial, taste, affective), while increasing reactivity to novel stimuli and modulating arousal-driven memory consolidation and reconsolidation (Decker *et al.*, 1990; Heron *et al.*, 1996; Cahill *et al.*, 2000; Clayton and Williams, 2000; Spreng *et al.*, 2001; Myers and Rinaman, 2002; Dębiec and Ledoux, 2004; Miranda *et al.*, 2008; Gazarini *et al.*, 2013; Giustino and Maren, 2018). SNS-related signaling further appears to regulate learning through trial-and-error (Amemiya *et al.*, 2016), which underscores how arousal-related neurobiological systems may drive learning through the accumulation of priors. More recently, parallel evidence has been observed in humans, wherein SNS-related signaling can alter learning and memory across many domains, including in the affective contexts of reward, threat, and uncertainty (Coull *et al.*, 1997; Kroes *et al.*, 2010; Mihov *et al.*, 2010; Soeter and Kindt, 2011; Marshall *et al.*, 2016; Chae *et al.*, 2019). For instance, recent evidence suggests that the same neurobiology also helps regulate prediction updating in humans during learning tasks (Jepma *et al.*, 2018).

In addition to arousal-related neurobiology supporting learning, these systems are firmly implicated in the computation of risk and resultant decisions and behaviors. Prior experiments suggest that pharmacologically attenuating SNS activation using beta-blockers such as propranolol to disrupt beta-adrenergic signaling can impair cognitive processes related to advantageous risk-taking. Yet, most of this work has been conducted in the context of chance-based gambling tasks. For example, individuals randomly assigned to take propranolol were less able to discriminate large potential losses and gains from small ones, in order to guide advantageous gambling decisions (Rogers *et al.*, 2004). Propranolol has also been shown to reduce the ability to track and refer to recent experiences (Lempert *et al.*, 2017), reduce aversion to monetary loss (Sokol-Hessner *et al.*, 2015b) and diminish amygdala-driven modulation of memory in contexts of chance (Phelps, 2006). Despite this work suggesting that beta-adrenergic signaling causally influences decision-making during chance-based gambling, we understand little about whether this same pathway impacts risk-taking during situations in which learning from experience is crucial for success.

## Present study

The present study thus used propranolol, a beta-blocker that blocks the SNS-related effects of epinephrine and norepinephrine at the sites of $\beta $1 and $\beta $2 adrenoceptors (Turner *et al.*, 1965) in order to blunt neurophysiological arousal. Specifically, in a randomized, double-blind, placebo-controlled mechanistic trial, participants took a single 40 mg dose of propranolol or a placebo and completed the BELT to examine beta-adrenergic impacts on learning and advantageous risk-taking. We hypothesized that individuals on propranolol (*vs* placebo) would learn the task parameters less effectively and thus take fewer advantageous risks, due to blunted access to neurophysiological arousal.

## Methods

### Participants

Data presented here were collected as part of a larger project examining how beta-adrenergic receptor blockade impacts reactivity to stress (MacCormack *et al.*, 2021; MacCormack *et al.,* in press). None of the data herein are published elsewhere. Participants were recruited from the University of North Carolina at Chapel Hill and its surrounding community via flyers, class announcements, and email listservs, and then screened for eligibility via telephone interview and an in-person visit. Individuals were excluded if they: reported prior/current use of beta-blockers, cigarettes, substances or prescription medications; had a history of/current physical or mental illness, a pacemaker, known cardiac irregularities, body mass index (BMI) over 33 or if they exhibited low resting diastolic blood pressure (DBP < 80 Hg/ml) or heart rate (HR < 60 bpm), given that low BP/HR are contraindications for propranolol. Of the 90 total participants enrolled in the study, three had missing BELT data due to computer error. The remaining 87 participants (45% female; *M*_age_ = 20.1 ± 1.46 years, 18–25 years; 56% White, 25% Asian, 9% Black, 7% bi- or multiracial and 2% other) are included herein, with *n = *42 randomly assigned to take propranolol and *n = *45 randomly assigned to take placebo. Drug groups were randomized such that they were matched on sex [*t*(85) = .074, *P = *0.942] and race/ethnicity [$\chi $^2^(4, *N = *87)= 1.25, *P=* 0.870]. See Table 1 for full participant characteristics and the supplementary materials (SMs) for details on statistical power.

**Table 1.** Participant characteristics

Demographics	Placebo	Propranolol	Total
Sex, *n* (%)			
Female	20 (23.0)	19 (21.8)	39 (44.8)
Male	25 (28.7)	23 (26.4)	48 (55.2)
Race, *n* (%)			
Asian descent	11 (12.6)	11 (12.6)	22 (25.3)
African descent	5 (5.7)	3 (3.4)	8 (9.2)
European descent	26 (30.0)	23 (26.4)	49 (56.3)
Bi- or multiracial	2 (2.3)	4 (4.6)	6 (6.9)
Other	1 (1.1)	1 (1.1)	2 (2.3)
Age, mean ± SD	20.49 ± 1.59	20.07 ± 1.30	20.28 ± 1.45
BMI, mean ± SD	22.96 ± 2.38	22.47 ± 2.52	22.72 ± 2.45
Objective socioeconomic status (SES), mean ± SD	16.48 ± 1.95	16.24 ± 1.88	16.36 ± 1.92

Frequency counts show percentages of total sample. The “Total” column for Age, BMI, and SES represents the mean and SD for the full sample. Objective SES was operationalized as the mean years of education that both parents completed. There were no significant differences between drug groups (as tested by Pearson’s chi-square and independent samples *t*-tests) in sex, race, age, BMI or objective SES (all *P* values *>* 0.10).

### Procedures

Participants received either a visually identical propranolol (40 mg) or placebo tablet, which they self-administered orally under supervision. A single propranolol dose of 40 mg was chosen given that higher doses may have lowered HR/BP to the point of causing fainting in our healthy, young adult sample, and given that 40 mg is a common clinical dosage administered for one-time performance anxiety situations (e.g. Currie *et al.*, 1988; Alexander *et al.*, 2007; Ernst *et al.*, 2016). Given that this was part of a larger study examining stress (see Open Science Framework (OSF)), all participants in both conditions first completed a standard laboratory paradigm designed to elicit social stress (Kirschbaum *et al.*, 1993), reported their affective responses, and provided biological samples. Two hours after completing the stressor and 3.5 h after ingesting the propranolol or placebo, participants completed the BELT. Given that the half-life of propranolol is 5 h after oral administration (Paterson *et al.*, 1970; Williams *et al.*, 1986), propranolol was still in effect during this task. Participants were compensated $100 USD and discharged after confirming that their HR and BP had returned to baseline levels. Procedures were approved by the University of North Carolina at Chapel Hill’s Human Subjects Protection Committee (IRB #16-2498).

### Measures

#### BELT.

To measure risk-taking and learning, participants completed the BELT, a computer task in which participants make decisions about how much to pump up three different colored balloons in order to obtain the highest score. Participants were told that the more points they earned in the game, the more money they would receive as an extra reward.

Participants pumped three different types of balloons that differed by color (blue, pink and orange). Each successful pump was worth one point regardless of balloon color, but each balloon color exploded after a different number of pumps. Specifically, certain-long balloons always exploded at 20 pumps, certain-short balloons always exploded at 8 pumps and uncertain balloons were unpredictable, exploding at 8, 14, or 20 pumps, depending on the trial. Participants were not told that balloon colors signified different explosion points, but they were explicitly told that not all balloons explode at the same point. Thus, to perform well on the task, participants needed to learn the strength of each balloon type (i.e. color). To make the most advantageous decisions on when to continue *vs* stop pumping, participants had to learn that certain-long balloons could be pumped the most and would yield the greatest number of points, certain-short balloons could only be pumped a few times and yielded fewer points but were still predictable, whereas uncertain balloons could sometimes be pumped many times and thus yield many points, but were risky because they would sometimes explode quickly.

To track learning effects, the BELT is divided into three separate task phases (Humphreys *et al.*, 2013). Participants first complete an early phase (first 1/3 of trials), wherein they know little about which balloons are the least *vs* most risky. This is when we would expect participants to experiment and learn through trial-and-error. The second or mid-phase allows individuals to continue learning and fine-tuning their risk predictions based on the early phase. Finally, the third or late phase is where individuals can most fully apply whatever information they gained from the prior phases (if they learned effectively) in order to make the most advantageous risk decisions. Ultimately, we expected that if individuals are effectively learning about risk throughout the task, then by the late phase, they should be at their most effective in judging when to *vs* not to pump up balloons further.

There were 18 trials per balloon type across the entire task (54 trials in total), and for each third of the task, there was an equal number of trials of each balloon type. This task was identical to that used in prior work (Humphreys *et al.*, 2013), except that we doubled the number of trials, allowing us to examine learning over a longer period of time and providing more opportunities for participants to explore and learn the different balloon contingencies. Participants pressed the spacebar to ‘pump up’ balloons. After the first pump, participants could press another button to ‘cash in’ their pumps for points, or they could continue pumping the balloons. Points accumulated across the course of the entire task. If participants pumped beyond a balloon’s limit, an explosion occurred, resulting in the loss of all points for that trial. We examined two primary outcomes from the BELT: (1) number of points, which served as our measure of overall task performance and (2) number of pumps, which served as our measure of risk-taking. Finally, as a secondary measure of risk-taking—and more specifically, untempered risk-taking, we examined (3) the number of explosions that an individual incurred. Given that we doubled the number of trials compared with prior work (e.g. Humphreys *et al.*, 2013), in analyses, we first replicated prior findings with this lengthened task in the placebo group to confirm that participants effectively learned task parameters (see SMs).

#### Covariates.

Both negative, high arousal affect post-stressor and BMI were examined as covariates, to assess whether the stressor from 2-h previously had any lingering effects on BELT performance, and whether BMI altered dosage effects of propranolol. There were no main effects or interactions of either covariate with propranolol or the BELT task parameters in predicting outcomes (see SMs for full details and results).

### Data analyses

Following prior analytical approaches with the BELT (Humphreys *et al.*, 2013), we examined task outcomes (i.e. points, pumps, explosions) by balloon type (i.e. certain-long, certain-short, uncertain) and by task phase (i.e. early, mid, late). Table 2 displays descriptive statistics for the BELT outcomes split by drug. We conducted three separate mixed ANOVAs (with points, pumps and explosions as the outcome, respectively), with balloon type (certain-long, certain-short, uncertain) × task phase (early, mid, late) as within-subjects predictors and drug (0 = placebo, 1 = propranolol) as a between-subjects predictor. Significant interactions were probed via ANOVAs within each specific task phase and balloon type, to minimize the inflation of a Type 1 error due to multiple pairwise comparison testing (Kao and Green, 2008). Results presented herein are the main effects of drug, and interactions of drug with the within-subjects variables (e.g. task phase, balloon type). Full results are presented in the SMs.

**Table 2.** Mean points, pumps and explosions by balloon condition, drug and task phase

			Task phase					
			Early phase		Mid-phase		Late phase	
Outcome	Balloon	Drug	*M*	SD	*M*	SD	*M*	SD
Points^a^	Certain-long	Placebo	55.02	18.34	66.98	23.53	73.69	27.54
		Propranolol	49.38	15.07	60.83	25.37	59.95	23.53
	Certain-short	Placebo	16.31	8.86	24.18	11.02	26.07	11.21
		Propranolol	16.14	6.57	19.57	9.81	22.14	11.63
	Uncertain	Placebo	37.07	13.35	35.40	13.17	39.29	11.25
		Propranolol	26.69	10.26	34.10	13.61	29.02	13.21
Pumps^b^	Certain-long	Placebo	59.60	21.94	71.44	27.49	79.13	29.93
		Propranolol	55.69	23.22	64.26	28.99	66.29	29.72
	Certain-short	Placebo	42.31	4.96	41.82	4.34	40.73	4.86
		Propranolol	40.19	7.03	40.52	7.25	39.90	7.25
	Uncertain	Placebo	48.53	12.02	48.53	12.03	49.89	10.34
		Propranolol	50.45	15.26	49.79	13.56	51.05	16.06
Explosions^b^	Certain-long	Placebo	0.33	0.56	0.38	0.65	0.42	0.69
		Propranolol	0.41	0.73	0.24	0.43	0.52	0.77
	Certain-short	Placebo	3.38	1.50	2.24	1.69	1.96	1.68
		Propranolol	3.07	1.40	2.69	1.81	2.38	1.96
	Uncertain	Placebo	1.67	1.07	1.56	1.39	1.20	1.31
		Propranolol	1.38	1.08	1.83	1.53	1.31	1.28

Points represent performance on the BELT, pumps represent risk-taking and explosions represent untempered risk-taking. The total task contained 54 trials, with 18 trials (six of each balloon type) in each phase of the task.

a In each task phase, participants could earn a maximum of 114 points from the certain-long balloons, 42 points from the certain-short balloons, and variable number of points from the uncertain balloons.

b In each task phase, the certain-long balloon exploded on the 20th pump (with a maximum number of 114 possible safe pumps and up to six explosions), the certain-short balloons exploded on the 8th pump (with a maximum number of 42 possible safe pumps and up to six explosions) and *uncertain* balloons exploded on the 8th, 14th or 20th pumps (with a variable number of maximum possible safe pumps and up to six explosions). For any trial where a balloon exploded, all points on that trial were lost.

After testing main effects of drug and interactions, we ran a mediation model using SPSS PROCESS Model 4 (Hayes, 2012), in order to model a simple mediation between (*a*) the predictor of drug (0 = placebo, 1 = propranolol), (*b*) the primary mediator of interest, pumps made in the late phase with the certain-long balloon, and (*c*) the primary outcome, total number of points overall achieved across the entire BELT. To assess the indirect effect (a*b), we used a nonparametric boot-strap procedure with replacement (*N* = 5000) with 95% bias-corrected confidence intervals (CIs). If the CIs did not include zero, the indirect effect was considered statistically significant.

## Results

### Beta-adrenergic blockade reduces overall task performance

**Fig. 1.**
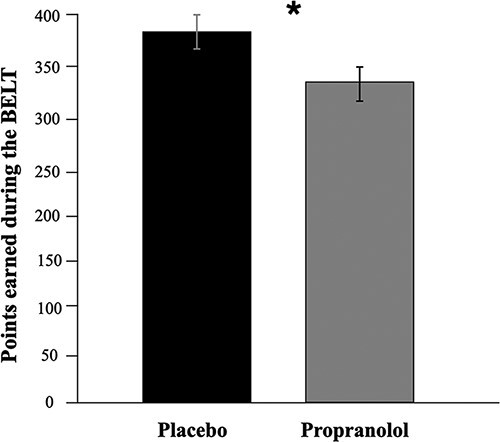
Points earned by placebo and propranolol groups across the task. There was a main effect of drug, *F*(1, 84) = 4.85, *P *= 0.030, partial *η*^2^ = 0.055, such that participants on propranolol (*M *= 337.74, SD* *= 72.50) earned fewer points across the task than those on placebo (*M *= 373.96, SD* *= 82.44). Error bars are standard errors.

To examine whether SNS signaling via beta-adrenergic receptors affects the overall ability to perform well on the BELT, we assessed the effects of propranolol on the number of points earned (Tables 3–4). As shown in Figure 1, there was a main effect of drug, *F*(1, 84) = 4.86, *P *= 0.030, partial *η*^2^ = 0.055, such that participants on propranolol (*M = *337.74, SD* *= 72.50) earned fewer points overall in the task overall relative to those on placebo (*M *= 373.96, SD* *= 82.44). This suggests that attenuated beta-adrenergic signaling impaired overall task performance. There were no two- or three-way interactions of task phase or balloon type with drug on total points earned across the task (see Table 3).

**Table 3.** Mixed effects ANOVAs assessing overall effects of propranolol, balloon type and BELT task phase on BELT points earned, pumps made and explosions, controlling for negative, high arousal affect

		Points model			Pumps model			Explosions model		
*Predictors*	*df*	*F*	*P*	*partial η^2^*	*F*	*P*	*partial η^2^*	*F*	*P*	*partial η^2^*
Between-subject effects										
Intercept	1	**224.34**	**0.000**	**0.728**	**192.41**	**0.000**	**0.699**	**56.63**	**0.000**	**0.403**
Drug	1	**4.86**	**0.030**	**0.055**	1.90	0.172	0.022	0.16	0.688	0.002
Affect	1	0.22	0.641	0.003	0.55	0.463	0.007	0.56	0.456	0.007
Error	84									
Within-subject effects										
Balloon	2	**35.26**	**0.000**	**0.296**	**11.94**	**0.000**	**0.124**	**20.43**	**0.000**	**0.196**
Balloon × Drug	2	2.65	0.092	0.031	2.22	0.135	0.026	0.23	0.728	0.003
Balloon × Affect	2	0.38	0.613	0.004	0.19	0.713	0.002	0.14	0.808	0.002
Balloon (Error)	168									
Task phase	2	3.08	0.051	0.035	2.36	0.115	0.027	0.70	0.491	0.008
Task phase × Drug	2	1.57	0.213	0.018	0.38	0.616	0.004	2.43	0.094	0.028
Task phase × Affect	2	0.01	0.984	0.000	0.40	0.599	0.005	0.88	0.412	0.010
Task phase (Error)	168									
Balloon × Task	4	**2.72**	**0.034**	**0.031**	**6.53**	**0.000**	**0.072**	**2.86**	**0.031**	**0.033**
Balloon × Task × Drug	4	1.32	0.264	0.015	**3.91**	**0.011**	**0.044**	1.52	0.203	0.018
Balloon × Task × Affect	4	0.70	0.584	0.008	0.78	0.500	0.009	1.40	0.239	0.016
Balloon × Task (Error)	336									

*Drug* was coded 0 = Placebo, 1 = Propranolol. Affect refers to post-stressor mean negative, high arousal affect. Balloon included three types: certain-long, certain-short and uncertain. Task included three phases or averaged timepoints: the early task phase, mid-task phase, and the late task phase. Given that the repeated measures of balloon type and task phase were significant in Mauchly’s test of sphericity (*P *< 0.000), model *P*-values reported use the Greenhouse–Geisser correction. Significant effects are bolded.

### Beta-adrenergic blockade reduces advantageous risk-taking

To examine how SNS signaling via beta-adrenergic receptors impacts advantageous risk-taking, we assessed the effects of propranolol on the number of pumps made. There was no main effect of drug, although participants on propranolol on average made fewer pumps overall (*M = *457.14, SD* *= 116.02) relative to those on placebo (*M *= 487.36, SD* *= 98.93). There was, however, a significant three-way interaction between drug, balloon type and task phase, *F*(4, 336) = 3.91, *P *= 0.011, partial *η*^2^ = 0.044 (see Table 3). To probe this interaction, we ran mixed ANOVAs within each task phase (early, mid, late) to examine the main effects and interaction of balloon type and drug. Within the late task phase (but not the early or mid-phases), we also found a significant interaction of balloon type × drug, *F*(2, 168) = 4.52, *P *= 0.027, partial *η*^2^ = 0.051 (see Table S1 in SMs).

**Fig. 2.**
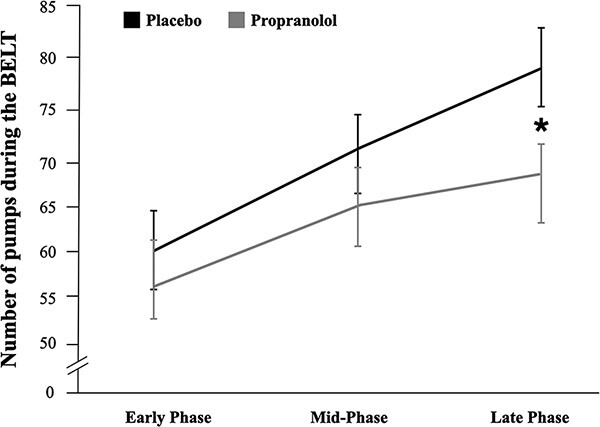
Pumps across task phases, split by drug group, within the certain-long balloon. The difference in pumps between drug groups was only significant for the certain-long balloon in the final phase of the task, *F*(1, 84) = 4.39, *P *= 0.039, *η*^2^ = 0.050, and not the first two task phases (*P *> 0.05), such that the propranolol group (*M *= 66.29, SD* *= 29.72) pumped less in the final third of the task than did the placebo group (*M *= 79.13, SD* *= 29.93). Error bars are standard errors.

To probe this interaction further, we conducted three separate ANOVAs within the late task phase for each balloon type (Table 4). In each of these three models, drug was the independent variable (i.e. between-subjects factor) and pumps in the late phase was the dependent variable. As shown in Figure 2, the difference in pumps in the late task phase between drug groups was only significant for the certain-long balloon (i.e. the balloon type with the greatest opportunity for advantageous risk-taking), *F*(1, 84) = 4.39, *P *= 0.039, *η*^2^ = 0.050. Specifically, individuals on propranolol pumped the certain-long balloon less (*M *= 66.29, SD* *= 29.72) than those on placebo (*M *= 79.13, SD* *= 29.93) in the late task phase. This effect of drug in the late task phase was not observed for the certain-short nor uncertain balloons.

**Table 4.** Univariate ANOVAs probing overall effects of propranolol on BELT pumps in the late task phase split by balloon type, controlling for negative, high arousal affect

		Long-certain balloon model			Short-certain balloon model			Uncertain balloon model		
*Predictors*	*df*	*F*	*P*	*η^2^*	*F*	*P*	*η^2^*	*F*	*P*	*η^2^*
Between-subject effects										
Intercept	1	**72.19**	**0.000**	**0.462**	**479.18**	**0.000**	**0.851**	**153.86**	**0.000**	**0.647**
Drug	1	**4.39**	**0.039**	**0.050**	0.58	0.448	0.007	0.11	0.744	0.001
Affect	1	0.61	0.438	0.007	0.92	0.339	0.011	0.22	0.639	0.003
Error	84									

Drug was coded 0 = Placebo, 1 = Propranolol. Affect refers to post-stressor mean negative, high arousal affect. Significant effects are bolded.

As a secondary measure of risk-taking—and more specifically, untempered risk-taking, we examined the number of explosions. Although the propranolol group exploded more balloons on average (*M = *13.07, SD* *= 6.32) than those on placebo (*M = *12.38, SD* *= 5.82), there was no main effect of drug, nor any two-way or three-way interactions between drug, balloon type and task phase (Table 3). These findings suggest that people in both the propranolol and placebo conditions exploded balloons at a similar rate.

### Mediation linking propranolol with reduced task performance

**Fig. 3.**
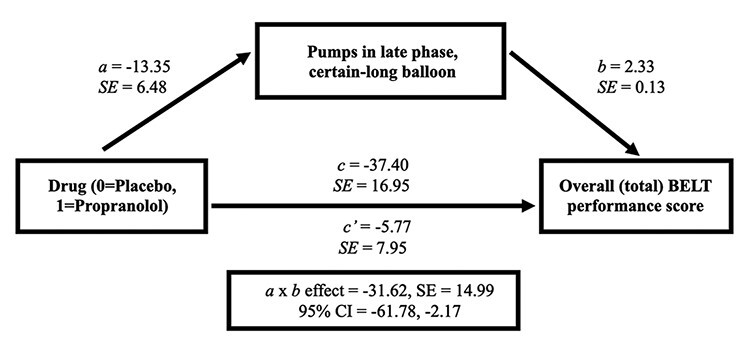
Mediation model. The link between drug group and higher overall BELT performance scores (total accumulated points across the entire task) was mediated via a significant indirect (a*b) effect of pumps made in the late phase with the certain-long balloon, estimated between 95% CIs [−61.78, −2.17]. Because zero was not within the 95% CI, the indirect effect was significantly different from zero at *P *< 0.05. Note all paths are significant but see “Results” for specific details.

Finally, in a mediation model, we examined if decreased pumping of the certain-long balloon during the final phase explained why individuals on propranolol scored fewer points overall relative to those on placebo. As shown in Figure 3, all paths were significant (*P *< 0.00–0.03), with a significant total effect (*c* = −37.40, SE = 16.95, *P* = 0.028). The indirect (a*b) effect was also significant, 95% CIs [−61.78, −2.17], demonstrating mediation. This suggests that blunted neurophysiological arousal (i.e. via SNS-related beta-adrenergic signaling) among those on propranolol disrupted optimal performance in part because it decreased effective learning about which risks were advantageous, particularly in the task condition with the most opportunity for risk-taking.

## Discussion

The goal of this study was to examine how in-the-moment neurophysiological arousal impacts learning which risks are likely to be rewarded *vs* detrimental. In a sample of healthy young adults, we pharmacologically manipulated SNS-related beta-adrenergic signaling, a key contributor to neurophysiological arousal, and examined consequent effects on risk-taking during a task in which learning from experience is critical for success. We found that individuals randomly assigned to take propranolol earned fewer points in the task than those on placebo, suggesting that blockade of beta-adrenergic signaling impaired performance. Moreover, mediation analysis suggested that attenuated beta-adrenergic signaling impaired performance in part because it reduced learning about which risks (i.e. balloon pumps) were advantageous. Together, these results suggest that a certain amount of neurophysiological arousal can help individuals more effectively learn over time which risks are advantageous, ultimately optimizing decision-making performance.

Specifically, we found that individuals with full access to their neurophysiological signals (i.e. those on a placebo) took more risks compared with those with attenuated neurophysiological arousal (i.e. those on propranolol), but only in the task condition that allowed the most risk-related exploration (i.e. the balloon that exploded the slowest) and only toward the end of the task (i.e. in the last phase of the trials). Indeed, mediation analysis showed that the placebo group’s greater pumping during this condition partially explained their greater overall task performance. We take these findings as evidence that SNS-related beta-adrenergic signaling helped facilitate more effective information gathering and risk-related learning, leading those on placebo to ultimately take more advantageous risks. In contrast, there were no arousal effects on BELT performance in the early phase, as presumably both groups (both placebo and propranolol) were gathering information about the risky nature of each balloon type. Likewise, there were no effects of propranolol in the mid-phase of the task, suggesting that beta-adrenergic facilitation of risk-related learning may take time to unfold. Beta-adrenergic signaling also did not impact risk-taking behavior within the certain-short balloon type (i.e. balloons that quickly exploded consistently). Indeed, it appears that all participants quickly mastered the meaning of certain-short balloons, perhaps because quickly exploding balloons may be more surprising or easy to detect. Similarly, beta-adrenergic signaling did not impact risk-taking in the uncertain condition, in which balloons exploded seemingly at random, consistent with the notion that arousal could not facilitate effective learning when there were no predictable rules or parameters that could be inferred from the context.

Collectively, these findings are consistent with classic work wherein an optimal amount of arousal can be facilitative for performance (Yerkes and Dodson, 1908) whereas too little arousal (e.g. when blunted by beta-blockade) can impair performance. We did not test what might happen when there is a high degree of neurophysiological arousal (e.g. on administration of epinephrine or norepinephrine), but it is likely that this would likewise impair performance on the BELT, given that high arousal states and related neurophysiology can impair several domains of performance, memory and cognition (e.g. Wichary *et al.*, 2016; Maran *et al.*, 2017; Marko and Riečanský, 2018).

One possible psychological mechanism underlying these findings is that optimal neurophysiological arousal heightens attention and salience of low-level perceptual cues that augment performance. Supporting this interpretation, affect-as-information theory and related work posit that arousal provides valuable insight in part by heightening attention to important environmental stimuli (Storbeck and Clore, 2008; Critchley and Garfinkel, 2018). Indeed, SNS-derived physiological activation is known to amplify the sensitivity of sensory modalities associated with vigilance, such as pupil dilation (Bradley *et al.*, 2008; Lempert *et al.*, 2017). Supporting this, a recent study found that propranolol led individuals to commit to an early decision in an information sampling task, rather than continue to gather more information (Hauser *et al.*, 2018). Moreover, there is emerging evidence that visceral afferent signals and interoceptive awareness thereof can more broadly enhance evaluations of risk and learning more generally (Sokol-Hessner *et al.*, 2009, 2015a; Kandasamy *et al.*, 2016; Pfeifer *et al.*, 2017). Given that neurophysiological arousal facilitates the saliency of and attention to low-level perceptual cues in the environment, an optimal amount of arousal could, as part of learning, increase attention to the success *vs* failure of past and ongoing risky decisions, thus guiding effective decision-making.

A second possible psychological mechanism is that individuals on propranolol may have been less cognitively alert compared with those on placebo, which may have reduced their capacity to learn from task feedback (e.g. explosions or tracking of point gains). As propranolol lowers heart rate and blood pressure and can contribute to feelings of lethargy (Ko, 2002), individuals on propranolol may have exerted less effort in the task. In future studies, one way to assess this ‘effort’ hypothesis would be to collect trial-by-trial reaction times, as these could provide implicit measures of participant effort and risky decision deliberation. Unfortunately, we did not collect reaction time data during the BELT and thus can only speculate that propranolol-induced lethargy and/or a lack of alertness could be one pathway contributing to these effects.

This study had limitations. Although propranolol’s bioavailability peaks 1 h after ingestion, we did not administer the BELT until 3.5 h after participants took the medication. Although this is within the 5-h half-life of propranolol (Paterson *et al.*, 1970; Williams *et al.*, 1986), our effects may have differed or been stronger if the BELT was completed when the effect of propranolol was at their peak. Furthermore, we did not collect physiological measures proximal to BELT completion, which would have provided further confirmation that propranolol was still active. It is also possible that the stress task completed as part of the larger study influenced the present results, although we controlled for post-stressor negative, high arousal affect in analyses to reduce this possibility. Future replications and extensions wherein the BELT is completed at the peak of propranolol bioavailability and without preceding tasks would provide a more precise estimate of the effect of propranolol on advantageous risk-taking. In addition, future research should clarify the extent to which laboratory-based tasks such as the BELT generalize to real-world contexts wherein optimal performance is contingent upon higher-stakes learning, such as in classroom, health, and personal finance settings.

In sum, the present study adds to the growing literature on the role of arousal and SNS-related beta-adrenergic signaling as a key neurophysiological pathway subserving successful risk-taking and learning. These findings are important given that real-world risk-taking is often predicated upon experiential, adaptive learning processes (e.g. using predictions gained through trial and error) that support optimal risk-related decisions (Denrell, 2007; Pleskac, 2008). To our knowledge, this constitutes the first known causal evidence in humans that neurophysiological arousal instantiated by beta-adrenergic signaling contributes to our ability to learn when to take advantageous risks that lead to desired outcomes. More generally, these findings contribute to the growing understanding that physiology influences cognitive and behavioral processes (e.g. Eisenberger *et al.*, 2017; Critchley and Garfinkel, 2018; MacCormack and Lindquist, 2018).

## Supplementary Material

nsab047_Supp
