# Supplementary material for: Neurophysiological contributors to advantageous risk-taking: an experimental psychopharmacological investigation
Source: Soc Cogn Affect Neurosci. 2021 Apr 16;16(9):926–36. doi: 10.1093/scan/nsab047 (PMC8421704; doi:10.1093/scan/nsab047)
Supplement: nsab047_Supp [file nsab047_supp.zip › suppl.docx]

**Supplementary Materials for: “Neurophysiological Contributors to Advantageous Risk-Taking: An Experimental Psychopharmacological Investigation” in *Social Cognitive & Affective Neuroscience***

**Supplementary Methods**

**Power Estimates**

*A priori* power analyses were conducted to determine sample size with respect to the study’s primary goal of investigating the effects of beta-blockade with propranolol on stress reactivity, but not with respect to secondary measures such as the BELT. However, there are two prior studies which similarly investigated the effects of propranolol on decision-making and risk-taking: Sokol-Hessner, Lackovic et al. (2015) with a final overall *N=*47 and Lempert et al. (2017) with a final *N=*37. Our sample of *N=*87 roughly doubles that of prior studies, increasing the power to detect a between-subjects effect of propranolol. Finally, the BELT task is a within-subjects, repeated-measures design with numerous trials for each balloon type and task phase, affording greater power to detect significant effects.

**Covariates**

One of the primary questions of interest in the broader study was propranolol’s effect on participants’ emotional responses to an acute psychological stressor, which was operationalized as a mean score of self-reported negative, high arousal affect after the stress task. Negative, high arousal affect was assessed with the expanded Positive and Negative Affect Schedule (e.g., endorsement of emotions such as anxious, embarrassed, stressed; Watson, Clark, & Tellegen, 1988) after the stressor. Participants in the propranolol condition reported somewhat lower negative, high arousal affect after the stressor (*M*=1.63, SD=.67) relative to those in the placebo condition (*M*=1.82, SD=.61), although this was not significant: *t*(85)=1.36, *p=*.179. The BELT task was administered two hours after the termination of the stressor, which should have provided ample time for recovery. Nonetheless, as one could argue that there may be lingering stressor effects on BELT performance, we included negative, high arousal affect as a covariate in the present statistical models (though there was never a significant effect of this variable in any model).

Finally, some prior relevant work with propranolol and risk-taking has found that BMI can moderate propranolol’s effects (e.g., Sokol-Hessner, Lackovic et al., 2015 and Lempert et al., 2017), especially in samples with a wide range in participant BMI. In our study, we specifically excluded individuals with high BMI (i.e., greater than 33), resulting in a restricted BMI range (BMI=18-28) and no significant *drug condition* differences by BMI. Nonetheless, to support completeness in the literature, we report exploratory analyses controlling for BMI in addition to negative, high arousal affect. No BMI effects were observed (see below).

While our primary analyses control for negative, high arousal affect and/or BMI, we also report the results from unadjusted analyses with no covariates included in models in Tables S4-S5, for the sake of future meta analyses.

**Supplementary Results**

**Assessing Potential Participant Unblinding**

We assessed whether participants were able to correctly guess whether they were on placebo or propranolol using a Pearson $\chi$^2^ test. Within the placebo group (*n=*45), 43 participants (95.6% on placebo) correctly guessed they were on placebo, with only 2 participants (4.4% on placebo) incorrectly guessing they might be on propranolol. Within the propranolol group (*n=*42), 14 participants (36.8% on propranolol) correctly guessed they were on propranolol, 24 participants (63.2%) incorrectly thought they were on placebo, with responses missing from 4 participants on propranolol. The overall $\chi$^2^ test was significant: $\chi$^2^(1, *N=*87)= 13.90, *p<*.001, largely driven by placebo participants correctly guessing they were on placebo. Notably, when examining within-guess type effects, there were no significant differences between groups in terms of guessing they were on placebo nor guessing they were on propranolol (*ps<*.05). Altogether, this suggests that by the end of the study session when we asked participants to guess their condition, individuals on placebo were likely unblinded, but individuals on propranolol largely remained blinded to their condition (i.e., unable to guess greater than chance within the condition). The potential unblinding in the placebo condition could be explained by participants’ lack of noticeable side effects, consistent with other work noting this as common in placebo groups (Kolahi, Bang, & Park, 2009; Park, Bang, & Cañette, 2008; Schulz, Chalmers, & Altman, 2002). The fact that most individuals on propranolol also thought they were on placebo further suggests that propranolol at 40mg has a subtle (i.e., less noticeable) dampening beta-adrenergic cardiovascular effect, which is ideal for drug effect studies where it is better for drug effects to be unobtrusive.

**Replication of BELT Performance in the Placebo Condition Only**

Our first goal was to test for replication of findings from the BELT (Humphreys et al., 2013) with our lengthened design. In order to do so, we first examined outcomes of interest (i.e., points, pumps) first among the placebo group only (*n*=45) and then in the full sample (*n*=87). We ran two separate repeated-measures ANOVAs (one with *points* as outcome, one with *pumps* as outcome), with *balloon type* (certain-short, uncertain, certain-long) and *task phase* (early, mid, late) as within-subject factors, within the placebo group only. We also controlled for negative, high arousal affect measured after the stress task.

**Points Earned.** For our measure of performance, points earned, there was a main effect of *balloon type, F*(2, 84)=15.12, *p*<.001 partial η^2^=.261, such that the most points were earned in the certain-long condition (*M*=195.69, *SD*=63.61), followed by the uncertain condition (*M*=111.76, *SD*=14.65), and then the certain-short condition (*M=*66.56, *SD*=26.91). Post-hoc analyses revealed that the difference in points earned between each of the balloon types were all significant from one another (*p*<.01). There was also a main effect of *task phase*, *F*(2, 84)=3.85, *p*=0.25, partial η^2^=.82, such that number of points earned in each phase increased linearly across the task, with the greatest number of points earned in the late phase (i.e., last third of trials) of the task (*M*=139.04, *SD*=34.71), followed by mid-phase (*M*=126.56, *SD*=35.30) and then the early phase (*M*=108.40, *SD*=26.42). The differences between each of these task phases was also significant, *p*<.01. These results indicate an improvement in performance (i.e., number of points earned) with greater task experience (i.e., each subsequent phase of the task), and replicate prior work by Humphreys et al. (2013).

**Pumps Made.** For our measure of risk-taking, pumps, there was a main effect of *balloon type*, *F*(2, 84)=4.30, *p*=.017, partial η^2^=.09, such that there were the greatest number of pumps on the certain-long balloons (*M*=210.18, *SD*=72.28), followed by uncertain balloons (*M*=152.31, *SD*=32.20), and then certain-short balloons (*M*=124.87, *SD*=11.03). Only the difference between pumps in the certain-long balloon and pumps in the certain-short balloon was significant, *p*<.05. A marginal main effect was also found for *task phase*, *F*(2, 84)=2.54, *p*=.085, partial η^2^=.06, such that pumps increased in the late phase of the task, with more pumps in the late phase (*M*=169.76, *SD*=35.18), compared to the mid-phase (*M*=161.80, *SD*=36.83) or the early phase (*M*=155.80, *SD*=38.10), *p*<.05. The difference between pumps in the early and mid- phases of the task was not significant (*p*=.18). There was also a significant *balloon type x task phase* interaction, *F*(4, 168)=3.90, *p*<.005, partial η^2^=.08. To further examine this interaction, we conducted three additional ANOVAs within each task phase with pumps in each balloon type included as a within-subject factor (repeated measures). The difference between *balloon types* was only significant in the final phase of the task, *F*(2,84)=6.00, *p*=.004, partial η^2^=.12, although it was also marginal for the mid-phase of the task, *F*(2,84)=4.30, *p*=.017, partial η^2^=.091. This indicates that pumping differences between the balloon types were most prominent in the last phase of the task, when participants had learned task parameters.

**Explosions**. For our measure of un-tempered risk-taking, explosions, there was a main effect of *balloon type, F*(2, 84)=13.08, *p*<.001 partial η^2^=.23, such that the most explosions occurred in the certain-short balloons (*M*=7.31, *SD*=4.11), followed by uncertain balloons (*M*=4.22, *SD*=2.30), and then certain-long balloons (*M*=0.84, *SD*=1.04). These were all significantly different from each other, *p*<.001. There was also a marginal main effect of *task phase*, *F*(2, 84)=2.61, *p*=.08 partial η^2^=.06, such that there were significantly more explosions in the early phase of the task (*M*=5.12, *SD*=2.12), compared to the mid-phase (*M*=3.91, *SD*=2.76) and compared to the late phase (*M*=3.33, *SD*=2.18), and a trend toward more explosions in the mid-phase compared to the late phase (*p*=.09). These results indicate a general pattern of reduction in explosions with greater task experience and mirror prior work by Humphreys et al. (2013).

Taken together, analyses examining the effect of *balloon type* and *task phase* on points and pumps suggest that participants within the placebo condition were able to learn the basic task parameters across the testing session, and that we replicate the results of Humphreys et al. (2013) in this doubled version of the original BELT task, as examined within the placebo group.

**BELT Main Effects and Interactions between Balloon Type and Task Phase**

As the main goal of the present study was to examine the effects of beta-adrenergic blockade on advantageous risk-taking and risk-related learning, results presented in the main text focus on the main effects of *drug condition* and interactions between *drug condition*, *balloon type*, and *task phase* on points, pumps, and explosions. Primary results control for the between-subject covariates of post-stressor negative, high arousal affect)—see **Table S1**. Exploratory results additionally controlling for BMI are presented in **Tables S2-S3**.

**Points Earned.** For our measure of performance, points, there was a main effect of *balloon type*, *F*(2, 168)=35.26, *p*<.0001, partial η^2^=.30, such that participants scored the most points with the certain-long balloon (*M*=183.36, *SD*=61.98), followed by the uncertain balloon (*M*=110.82, SD=17.20), and then the certain-short balloon (*M*=62.36, *SD*=24.61). The differences between points earned across balloon types were all significantly different from each other, as revealed by post-hoc pairwise comparisons: long-certain vs. short-certain balloon, *M_diff_*= 40.23, *SE*=2.21, *p<*.0001, long-certain vs. uncertain balloon, *M_diff_*= 24.04, *SE*=1.97, *p<*.0001, and short-certain vs. uncertain balloon, *M_diff_*= -16.19, *SE*=1.13, *p<*.0001.

There was also a marginal effect of task phase, *F*(2, 168)=3.08, *p*=.051, partial η^2^=.04, such that points somewhat increased linearly across the task, with the greatest number of points in the late phase of the task (*M*=130.38, *SD*=35.34), followed by mid-phase (*M*=120.74, *SD*=34.59) and then the early phase (*M*=105.41, *SD*=23.87). The differences between each of these task phases were significant: early vs. mid-task, *M_diff_*= -5.07, *SE*=1.09, *p<*.0001, early vs. late task, *M_diff_*= -8.26, *SE*=1.18, *p<*.0001, and mid- vs. late task *M_diff_*= -3.18, *SE*=.97, *p=*.002. There was also a significant *balloon type x task phase* interaction for points, *F*(4, 336)=2.72, *p*=.034, partial η^2^=.03.

To further examine this *balloon type x task phase* interaction, we conducted additional ANOVAs separately for each *task phase*, with *balloon type* as a repeated-measures factor. In the early phase of the task, there was a significant effect of *balloon type*, *F*(2,168)=24.99, *p*<.0001, partial η^2^=.23. Post-hoc pair-wise comparisons showed that, in the early phase, participants scored the most points in the long condition, followed by the uncertain condition, and then the short condition, which were all significantly different from each other: long-certain vs. short-certain balloon, *M_diff_*= 35.97, *SE*=2.08, *p<*.0001, long-certain vs. uncertain balloon, *M_diff_*= 15.32, *SE*=1.79, *p<*.0001, and short-certain vs. uncertain balloon, *M_diff_*= -20.65, *SE*=1.70, *p<*.0001. In the mid-phase of the task, there was a similar significant effect of balloon typ,e *F*(2,168)=24.67, *p*<.0001, partial η^2^=.23, with the same pattern of participant scoring: long-certain vs. short-certain balloon, *M_diff_*= 42.02, *SE*=2.74, *p<*.0001, long-certain vs. uncertain balloon, *M_diff_*= 29.14, *SE*=2.53, *p<*.0001, and short-certain vs. uncertain balloon, *M_diff_*= -12.88, *SE*=1.80, *p<*.0001. Finally, there was the same effect of balloon type in the last phase of the task as well, *F*(2,168)=22.25, *p*<.0001, partial η^2^=.21, with the same pattern of participant scoring: long-certain vs. short-certain balloon, *M_diff_*= 42.69, *SE*=2.67, *p<*.0001, long-certain vs. uncertain balloon, *M_diff_*= 27.65, *SE*=2.90, *p<*.0001, and short-certain vs. uncertain balloon, *M_diff_*= -15.04, *SE*=1.85, *p<*.0001.

**Pumps Made.** For our measure of risk-taking, pumps, we broadly replicated our main findings from the placebo group analysis. There was again a main effect of *balloon type, F*(2, 168)=11.94, *p*<.0001, partial η^2^=.12, such that there were the greatest number of pumps on the certain-long balloons (*M*=198.62, *SD*=74.92), followed by uncertain balloons (*M*=151.80, *SD*=34.50) and then certain-short balloons (*M*=122.82, *SD*=16.39). The differences between pumps made across balloon types were all significantly different from each other, as revealed by post-hoc pairwise comparisons: long-certain vs. short-certain balloon, *M_diff_*= 25.15, *SE*=2.57, *p<*.0001, long-certain vs. uncertain balloon, *M_diff_*= 15.47, *SE*=2.14, *p<*.0001, and short-certain vs. uncertain balloon *M_diff_*= -9.68, *SE*=1.00, *p<*.0001. There was no main effect of *task phase, F*(2, 168)=2.36, *p*=.115, partial η^2^=.03. There was however, as above with points, a significant *balloon type x task phase* interaction for pumps, *F*(4, 336)=6.53, *p*<.0001, partial η^2^=.07.

To further examine this interaction, we conducted additional ANOVAs separately for each *task phase*, with *balloon type* as a repeated-measures factor. Post-hoc pairwise comparisons revealed that the interaction was driven by a strong increase in pumps made across the certain-long balloon type compared to stable pumps during the certain-short condition and the uncertain balloon type. Specifically, in the early phase, there was a significant effect of balloon type, *F*(2,168)=5.82, *p*=.009, partial η^2^=.07. Post-hoc pairwise comparisons revealed that participants pumped more in the long condition, followed by the uncertain condition, and then the short condition, which were all significantly different from each other: long-certain vs. short-certain balloon, *M_diff_*= 16.40, *SE*=2.33, *p<*.0001, long-certain vs. uncertain balloon, *M_diff_*= 20.77, *SE*=2.35, *p<*.0001, and short-certain vs. uncertain balloon, *M_diff_*= 4.37, *SE*=1.23, *p=*.001. In the mid-phase of the task, there was a similar significant effect of balloon type, *F*(2,168)=18.30, *p*<.0001, partial η^2^=.18, with the same pattern of pumps: long-certain vs. short-certain balloon, *M_diff_*= 26.70, *SE*=2.89, *p<*.0001, long-certain vs. uncertain balloon, *M_diff_*= 33.09, *SE*=2.88, *p<*.0001, and short-certain vs. uncertain balloon, *M_diff_*= 6.42, *SE*=1.55, *p<*.0001. Finally, there was the same effect of balloon type in the last phase of the task as well, *F*(2,168)=13.09, *p*<.0001, partial η^2^=.14, with a similar pattern of pumps as above: long-certain vs. short-certain balloon, *M_diff_*= 32.38, *SE*=3.22, *p<*.0001, long-certain vs. uncertain balloon, *M_diff_*= 22.23, *SE*=2.83, *p<*.0001, and short-certain vs. uncertain balloon, *M_diff_*= -10.15, *SE*=1.35, *p<*.0001.

**Explosions.** For explosions, there was a main effect of *balloon type, F*(2, 168)=20.43, *p*<.0001, partial η^2^=.20, such that the most explosions occurred in the certain-short balloons (*M*=7.59, *SD*=4.17), followed by uncertain balloons (*M*=4.28, *SD*=2.51), and then certain-long balloons (*M*=0.85, *SD*=1.13), *p*=.001. The differences between pumps made across balloon types were all significantly different from each other, as revealed by post-hoc pairwise comparisons: long-certain vs. short-certain balloon, *M_diff_*= -2.24, *SE*=.16, *p<*.0001, long-certain vs. uncertain balloon *M_diff_*= -1.11, *SE*=.09, *p<*.0001, and short-certain vs. uncertain balloon, *M_diff_*= 1.13, *SE*=.14, *p<*.0001. However, there was no effect of *task phase, F*(2, 168)=.70, *p*=.491, partial η^2^=.01. Finally, as in the other two models, there was a significant *balloon type x task phase* interaction, *F*(4, 336)=2.86, *p*=.031 partial η^2^=.03.

To further examine this interaction, we conducted additional ANOVAs to examine explosions in *balloon type* included as factors (repeated measures) within each task phase. Specifically, in the early phase, there was a significant effect of balloon type on explosions: *F*(2,168)=24.84, *p*<.0001, partial η^2^=.23. Post-hoc pairwise comparisons revealed that participants exploded balloons the least in the long condition, followed by the uncertain condition, and then the short condition, which were all significantly different from each other: long-certain vs. short-certain balloon, *M_diff_*= -2.86, *SE*=.16, *p<*.0001, long-certain vs. uncertain balloon, *M_diff_*= -1.16, *SE*=.11, *p<*.0001, and short-certain vs. uncertain balloon *M_diff_*= 1.70, *SE*=.17, *p<*.0001. In the mid-phase of the task, there was a similar significant effect of balloon type *F*(2,168)=9.64, *p*<.0001, partial η^2^=.10, with the same pattern of explosions as above: long-certain vs. short-certain balloon, *M_diff_*= -2.16, *SE*=.19, *p<*.0001, long-certain vs. uncertain balloon, *M_diff_*= -1.39, *SE*=.16, *p<*.0001, and short-certain vs. uncertain balloon, *M_diff_*= .77, *SE*=.18, *p<*.0001. Finally, there was the same effect of balloon type in the last phase of the task as well, *F*(2,168)=7.46, *p*=.002, partial η^2^=.08, with the same pattern of explosions as above: long-certain vs. short-certain balloon, *M_diff_*= -1.69, *SE*=.22, *p<*.0001, long-certain vs. uncertain balloon, *M_diff_*= -.78, *SE*=.16, *p<*.0001, and short-certain vs. uncertain balloon, *M_diff_*= .91, *SE*=.22, *p<*.0001.These results again indicate a reduction of explosions with greater task experience, particularly in the certain-short and uncertain conditions, and mirror our results in the placebo group and prior work (Humphreys et al., 2013).

**Supplementary Discussion**

Although not the focus of the present study, it is worth speculating about the possible neural pathways through which neurophysiological arousal may contribute to successfully learning what risks are likely to be rewarded vs. those that should be avoided. Prior research suggests that an amygdala-striatal circuit is important for guiding affect-based decision-making (van Holstein, MacLeod, & Floresco, 2020; Watanabe, Sakagami, & Haruno, 2013) and that propranolol attenuates amygdala activity (Hurlemann et al., 2010), suggesting the possibility that propranolol may disrupt effective risk-taking in part by blunting amygdala activity and/or amygdala-striatal connectivity (Phelps, Lempert, & Sokol-Hessner, 2014). Further, the anterior insula is known to play a critical role in integrating afferent physiological information to guide effective behavior (Craig, 2004, 2009; Critchley, 2009) while also helping identify salient stimuli that can then facilitate motivated behaviors (Uddin, 2015). Thus, diminished beta-adrenergic signaling among those on propranolol could potentially lead to both blunted amygdala and anterior insula activity and a corresponding decreased ability to track and identify optimal risk-taking conditions, a hypothesis that should be more fully tested in future studies. Alternatively, given that propranolol has been shown to disrupt the acquisition of emotion-related memories (Chalkia, Weermeijer, Van Oudenhove, & Beckers, 2019; Weymar et al., 2010; see Lonergan, Olivera-Figueroa, Pitman, & Brunet, 2013 for meta-analysis), it could be the case that individuals on propranolol did not learn as effectively which task conditions were optimally “risky” in part because they did not fully encode or update this information in memory during the task due to attenuations of amygdala and/or hippocampal activation. Future neuroimaging work that examines the effects of propranolol on the neural circuitry engaged during learning and risk-taking is needed to adjudicate between these different possibilities.

**Supplementary References**

Chalkia, A., Weermeijer, J., Van Oudenhove, L., & Beckers, T. (2019). Acute but not permanent effects of propranolol on fear memory expression in humans. *Frontiers in Human Neuroscience*, *13*, 51. https://doi.org/10.3389/fnhum.2019.00051

Craig, A. D. (2004). Human feelings: why are some more aware than others? *Trends in Cognitive Sciences*, *8*(6), 239–241. https://doi.org/10.1016/j.tics.2004.04.004

Craig, A. D. (2009). How do you feel — now? The anterior insula and human awareness. *Nature Reviews Neuroscience*, *10*, 59–70. https://doi.org/10.1038/nrn2555

Critchley, H. D. (2009). Psychophysiology of neural, cognitive and affective integration: fMRI and autonomic indicants. *International Journal of Psychophysiology : Official Journal of the International Organization of Psychophysiology*, *73*(2), 88–94. https://doi.org/10.1016/j.ijpsycho.2009.01.012

Hurlemann, R., Patin, A., Onur, O. A., Cohen, M. X., Baumgartner, T., Metzler, S., … Kendrick, K. M. (2010). Oxytocin enhances amygdala-dependent, socially reinforced learning and emotional empathy in humans. *Journal of Neuroscience*, *30*, 4999–5007. https://doi.org/10.1523/JNEUROSCI.5538-09.2010

Kolahi, J., Bang, H., & Park, J. (2009). Towards a proposal for assessment of blinding success in clinical trials: Up-to-date review. *Community Dentistry and Oral Epidemiology*, *37*, 477–484. https://doi.org/10.1111/j.1600-0528.2009.00494.x

Lempert, K. M., Lackovic, S. F., Tobe, R. H., Glimcher, P. W., & Phelps, E. A. (2017). Propranolol reduces reference-dependence in intertemporal choice. *Social Cognitive and Affective Neuroscience*, *12*, 1394–1401. https://doi.org/10.1093/scan/nsx081

Lonergan, M., Olivera-Figueroa, L., Pitman, R., & Brunet, A. (2013). Propranolol’s effects on the consolidation and reconsolidation of long-term emotional memory in healthy participants: a meta-analysis. *Journal of Psychiatry & Neuroscience*, *38*, 222–231. https://doi.org/10.1503/jpn.120111

Park, J., Bang, H., & Cañette, I. (2008). Blinding in clinical trials, time to do it better. *Complementary Therapies in Medicine*, *16*, 121–123. https://doi.org/10.1016/j.ctim.2008.05.001

Phelps, E. A., Lempert, K. M., & Sokol-Hessner, P. (2014). Emotion and decision making: Multiple modulatory neural circuits. *Annual Review of Neuroscience*, *37*, 263–287. https://doi.org/10.1146/annurev-neuro-071013-014119

Schulz, K. F., Chalmers, I., & Altman, D. G. (2002). The landscape and lexicon of blinding in randomized trials. *Annals of Internal Medicine*, *136*, 254. https://doi.org/10.7326/0003-4819-136-3-200202050-00022

Sokol-Hessner, P., Lackovic, S. F., Tobe, R. H., Camerer, C. F., Leventhal, B. L., & Phelps, E. A. (2015). Determinants of propranolol’s selective effect on loss aversion. *Psychological Science*, *26*, 1123–1130. https://doi.org/10.1177/0956797615582026

Uddin, L. Q. (2015). Salience processing and insular cortical function and dysfunction. *Nature Reviews Neuroscience*, *16*, 55–61. https://doi.org/10.1038/nrn3857

van Holstein, M., MacLeod, P. E., & Floresco, S. B. (2020). Basolateral amygdala – nucleus accumbens circuitry regulates optimal cue-guided risk/reward decision making. *Progress in Neuro-Psychopharmacology and Biological Psychiatry*, *98*, 109830. https://doi.org/10.1016/j.pnpbp.2019.109830

Watanabe, N., Sakagami, M., & Haruno, M. (2013). Reward prediction error signal enhanced by striatum-amygdala interaction explains the acceleration of probabilistic reward learning by emotion. *Journal of Neuroscience*, *33*, 4487–4493. https://doi.org/10.1523/JNEUROSCI.3400-12.2013

Weymar, M., Löw, A., Modess, C., Engel, G., Gründling, M., Petersmann, A., … Hamm, A. O. (2010). Propranolol selectively blocks the enhanced parietal old/new effect during long-term recollection of unpleasant pictures: A high density ERP study. *NeuroImage*, *49*, 2800–2806. https://doi.org/10.1016/j.neuroimage.2009.10.025

**Table S1.** ANOVAs probing effects of propranolol on BELT points, pumps, and explosions, split by task phase, controlling for negative, high arousal affect.

|  |  | **Early Task Phase** | | |  | **Mid-Task Phase** | | |  | **Late Task Phase** | | |
| --- | --- | --- | --- | --- | --- | --- | --- | --- | --- | --- | --- | --- |
| ***Predictors*** | ***df*** | ***F*** | ***p*** | ***partial η^2^*** |  | ***F*** | ***p*** | ***partial η^2^*** |  | ***F*** | ***p*** | ***partial η^2^*** |
| **BELT Points** |  |  |  |  |  |  |  |  |  |  |  |  |
| ***Between-subject effects*** |  |  |  |  |  |  |  |  |  |  |  |  |
| Intercept | 1 | **214.73** | **.000** | **.719** |  | **132.31** | **.000** | **.612** |  | **150.82** | **.000** | **.642** |
| Drug | 1 | 1.65 | .203 | .019 |  | 2.77 | .100 | .032 |  | **5.93** | **.017** | **.066** |
| Affect | 1 | 0.38 | .538 | .005 |  | 0.12 | .729 | .001 |  | 0.08 | .778 | .001 |
| Error | 84 |  |  |  |  |  |  |  |  |  |  |  |
| ***Within-subject effects*** |  |  |  |  |  |  |  |  |  |  |  |  |
| Balloon | 2 | **24.99** | **.000** | **.229** |  | **24.67** | **.000** | **.227** |  | **22.25** | **.000** | **.209** |
| Balloon x Drug | 2 | 1.44 | .239 | .017 |  | 0.71 | .468 | .008 |  | **3.91** | **.030** | **.044** |
| Balloon x Affect | 2 | 0.11 | .884 | .001 |  | 0.73 | .460 | .009 |  | 0.49 | .578 | .007 |
| Balloon (Error) | 168 |  |  |  |  |  |  |  |  |  |  |  |
|  |  |  |  |  |  |  |  |  |  |  |  |  |
| **BELT Pumps** |  |  |  |  |  |  |  |  |  |  |  |  |
| ***Between-subject effects*** |  |  |  |  |  |  |  |  |  |  |  |  |
| Intercept | 1 | **195.37** | **.000** | **.699** |  | **160.01** | **.000** | **.656** |  | **194.84** | **.000** | **.699** |
| Drug | 1 | 0.96 | .330 | .011 |  | 1.60 | .209 | .019 |  | 2.50 | .118 | .029 |
| Affect | 1 | 0.11 | .747 | .001 |  | 0.19 | .668 | .002 |  | 0.80 | .374 | .009 |
| Error | 84 |  |  |  |  |  |  |  |  |  |  |  |
| ***Within-subject effects*** |  |  |  |  |  |  |  |  |  |  |  |  |
| Balloon | 2 | **5.83** | **.009** | **.065** |  | **18.30** | **.000** | **.179** |  | **13.09** | **.000** | **.135** |
| Balloon x Drug | 2 | 0.33 | .642 | .004 |  | 1.16 | .304 | .014 |  | **4.52** | **.027** | **.051** |
| Balloon x Affect | 2 | 0.04 | .914 | .000 |  | 0.95 | .363 | .011 |  | 0.34 | .615 | .004 |
| Balloon (Error) | 168 |  |  |  |  |  |  |  |  |  |  |  |
|  |  |  |  |  |  |  |  |  |  |  |  |  |
| **BELT Explosions** |  |  |  |  |  |  |  |  |  |  |  |  |
| ***Between-subject effects*** |  |  |  |  |  |  |  |  |  |  |  |  |
| Intercept | 1 | **47.66** | **.000** | **.362** |  | **35.72** | **.000** | **.298** |  | **33.75** | **.000** | **.287** |
| Drug | 1 | 1.03 | .314 | .012 |  | 0.61 | .436 | .007 |  | 1.18 | .280 | .014 |
| Affect | 1 | 0.01 | .919 | .000 |  | 1.15 | .287 | .014 |  | 0.59 | .443 | .007 |
| Error | 84 |  |  |  |  |  |  |  |  |  |  |  |
| ***Within-subject effects*** |  |  |  |  |  |  |  |  |  |  |  |  |
| Balloon | 2 | **24.84** | **.000** | **.228** |  | **9.64** | **.000** | **.103** |  | **7.46** | **.002** | **.082** |
| Balloon x Drug | 2 | 1.06 | .342 | .012 |  | 1.41 | .248 | .016 |  | 0.26 | .734 | .003 |
| Balloon x Affect | 2 | 0.12 | .855 | .001 |  | 1.03 | .356 | .012 |  | 0.81 | .429 | .010 |
| Balloon (Error) | 168 |  |  |  |  |  |  |  |  |  |  |  |

**Note**: *Drug* was coded 0=Placebo, 1=Propranolol. *Affect* refers to post-stressor mean negative, high arousal affect. Given that *balloon type* was significant in Mauchly’s test of sphericity, model *p*-values reported use the Greenhouse-Geisser correction. Significant effects are bolded.

**Table S2.** Mixed effects ANOVAs assessing overall effects of propranolol, balloon type, and BELT task phase on BELT points earned, pumps made, and explosions, controlling for both affect and BMI.

|  |  | **Points Model** | | |  | **Pumps Model** | | |  | **Explosions Model** | | |
| --- | --- | --- | --- | --- | --- | --- | --- | --- | --- | --- | --- | --- |
| ***Predictors*** | ***df*** | ***F*** | ***p*** | ***partial η^2^*** |  | ***F*** | ***p*** | ***partial η^2^*** |  | ***F*** | ***p*** | ***partial η^2^*** |
| ***Between-subject effects*** |  |  |  |  |  |  |  |  |  |  |  |  |
| Intercept | 1 | **197.33** | **.000** | **.704** |  | **192.41** | **.000** | **.699** |  | **49.55** | **.000** | **.374** |
| Drug | 1 | **4.96** | **.029** | **.056** |  | 1.90 | .172 | .022 |  | 0.15 | .699 | .002 |
| Affect | 1 | 0.16 | .686 | .002 |  | 0.55 | .463 | .007 |  | 0.51 | .476 | .006 |
| BMI | 1 | 0.79 | .377 | .009 |  | 0.36 | .553 | .004 |  | 0.18 | .676 | .002 |
| Error | 83 |  |  |  |  |  |  |  |  |  |  |  |
|  |  |  |  |  |  |  |  |  |  |  |  |  |
| ***Within-subject effects*** |  |  |  |  |  |  |  |  |  |  |  |  |
| Balloon | 2 | **28.81** | **.000** | **.258** |  | **9.34** | **.002** | **.101** |  | **17.89** | **.000** | **.177** |
| Balloon x Drug | 2 | 2.75 | .085 | .032 |  | 2.35 | .123 | .028 |  | 0.21 | .744 | .002 |
| Balloon x Affect | 2 | 0.29 | .671 | .004 |  | 0.14 | .755 | .002 |  | 0.13 | .811 | .002 |
| Balloon x BMI | 2 | 1.22 | .289 | .014 |  | 1.61 | .209 | .019 |  | 1.13 | .312 | .013 |
| Balloon (Error) | 166 |  |  |  |  |  |  |  |  |  |  |  |
|  |  |  |  |  |  |  |  |  |  |  |  |  |
| Task phase | 2 | 2.55 | .084 | .030 |  | 1.07 | .329 | .013 |  | 0.87 | .419 | .010 |
| Task phase x Drug | 2 | 1.57 | .212 | .019 |  | 0.40 | .603 | .005 |  | 2.33 | .104 | .027 |
| Task phase x Affect | 2 | 0.02 | .979 | .000 |  | 0.28 | .683 | .003 |  | 0.71 | .488 | .008 |
| Task phase x BMI | 2 | 0.10 | .899 | .001 |  | 2.68 | .090 | .031 |  | 2.44 | .093 | .029 |
| Task phase (Error) | 166 |  |  |  |  |  |  |  |  |  |  |  |
|  |  |  |  |  |  |  |  |  |  |  |  |  |
| Balloon x Task | 4 | 1.85 | .126 | .022 |  | **4.47** | **.005** | **.051** |  | 2.18 | .081 | .026 |
| Balloon x Task x Drug | 4 | 1.32 | .263 | .016 |  | **4.10** | **.009** | **.047** |  | 1.51 | .207 | .018 |
| Balloon x Task x Affect | 4 | 0.59 | .654 | .007 |  | 0.66 | .571 | .008 |  | 1.29 | .277 | .015 |
| Balloon x Task x BMI | 4 | 1.38 | .244 | .016 |  | 2.20 | .093 | .026 |  | 0.66 | .597 | .008 |
| Balloon x Task (Error) | 332 |  |  |  |  |  |  |  |  |  |  |  |

**Note**: *Drug* was coded 0=Placebo, 1=Propranolol. *Affect* refers to post-stressor mean negative, high arousal affect. *BMI* refers to body mass index. *Balloon* included three types: certain-long, certain-short, and uncertain. *Task* included three phases or averaged timepoints: the early task phase, mid-task phase, and the last or final task phase. Given that the repeated measures of *balloon type* and *task phase* were significant in Mauchly’s test of sphericity (*ps*<.000), model p-values reported use the Greenhouse-Geisser correction. Significant effects are bolded.

**Table S3.** ANOVAs probing effects of propranolol on BELT points, pumps, and explosions, split by task phase, controlling for both affect and BMI.

|  |  | **Early Task Phase** | | |  | **Mid-Task Phase** | | |  | **Late Task Phase** | | |
| --- | --- | --- | --- | --- | --- | --- | --- | --- | --- | --- | --- | --- |
| ***Predictors*** | ***df*** | ***F*** | ***p*** | ***partial η^2^*** |  | ***F*** | ***p*** | ***partial η^2^*** |  | ***F*** | ***p*** | ***partial η^2^*** |
| **BELT Points** |  |  |  |  |  |  |  |  |  |  |  |  |
| ***Between-subject effects*** |  |  |  |  |  |  |  |  |  |  |  |  |
| Intercept | 1 | **189.10** | **.000** | **.695** |  | **116.47** | **.000** | **.584** |  | **131.57** | **.000** | **.613** |
| Drug | 1 | 1.70 | .195 | .020 |  | 2.81 | .098 | .033 |  | **6.05** | **.016** | **.068** |
| Affect | 1 | 0.32 | .576 | .004 |  | 0.09 | .760 | .001 |  | 0.05 | .825 | .001 |
| BMI | 1 | 0.64 | .427 | .008 |  | 0.32 | .575 | .004 |  | 0.80 | .374 | .010 |
| Error | 83 |  |  |  |  |  |  |  |  |  |  |  |
| ***Within-subject effects*** |  |  |  |  |  |  |  |  |  |  |  |  |
| Balloon | 2 | **22.35** | **.000** | **.212** |  | **19.51** | **.000** | **.190** |  | **17.37** | **.000** | **.173** |
| Balloon x Drug | 2 | 1.44 | .239 | .017 |  | 0.77 | .443 | .009 |  | **4.03** | **.028** | **.046** |
| Balloon x Affect | 2 | 0.11 | .888 | .001 |  | 0.61 | .520 | .007 |  | 0.38 | .642 | .005 |
| Balloon x BMI | 2 | 0.09 | .906 | .001 |  | 1.55 | .218 | .018 |  | 1.69 | .194 | .020 |
| Balloon (Error) | 166 |  |  |  |  |  |  |  |  |  |  |  |
|  |  |  |  |  |  |  |  |  |  |  |  |  |
| **BELT Pumps** |  |  |  |  |  |  |  |  |  |  |  |  |
| ***Between-subject effects*** |  |  |  |  |  |  |  |  |  |  |  |  |
| Intercept | 1 | **175.27** | **.000** | **.679** |  | **139.63** | **.000** | **.627** |  | **170.46** | **.000** | **.673** |
| Drug | 1 | 0.96 | .331 | .011 |  | 1.67 | .199 | .020 |  | 2.60 | .111 | .030 |
| Affect | 1 | 0.10 | .758 | .001 |  | 0.13 | .715 | .002 |  | 0.68 | .413 | .008 |
| BMI | 1 | 0.03 | .859 | .000 |  | 0.87 | .354 | .010 |  | 1.03 | .314 | .012 |
| Error | 83 |  |  |  |  |  |  |  |  |  |  |  |
| ***Within-subject effects*** |  |  |  |  |  |  |  |  |  |  |  |  |
| Balloon | 2 | **5.54** | **.011** | **.063** |  | **14.10** | **.000** | **.145** |  | **9.33** | **.001** | **.101** |
| Balloon x Drug | 2 | 0.32 | .648 | .004 |  | 1.24 | .283 | .015 |  | **4.81** | **.022** | **.055** |
| Balloon x Affect | 2 | 0.04 | .922 | .000 |  | 0.80 | .416 | .010 |  | 0.23 | .694 | .003 |
| Balloon x BMI | 2 | 0.05 | .896 | .001 |  | 1.46 | .235 | .017 |  | 2.50 | .108 | .029 |
| Balloon (Error) | 166 |  |  |  |  |  |  |  |  |  |  |  |
|  |  |  |  |  |  |  |  |  |  |  |  |  |
| **BELT Explosions** |  |  |  |  |  |  |  |  |  |  |  |  |
| ***Between-subject effects*** |  |  |  |  |  |  |  |  |  |  |  |  |
| Intercept | 1 | **47.40** | **.000** | **.364** |  | **29.39** | **.000** | **.262** |  | **28.13** | **.000** | **.253** |
| Drug | 1 | 0.97 | .328 | .012 |  | 0.56 | .455 | .007 |  | 1.12 | .292 | .013 |
| Affect | 1 | 0.00 | .972 | .000 |  | 1.00 | .320 | .012 |  | 0.51 | .479 | .006 |
| BMI | 1 | 0.95 | .333 | .011 |  | 1.02 | .315 | .012 |  | 0.69 | .409 | .008 |
| Error | 83 |  |  |  |  |  |  |  |  |  |  |  |
| ***Within-subject effects*** |  |  |  |  |  |  |  |  |  |  |  |  |
| Balloon | 2 | **21.52** | **.000** | **.206** |  | **8.49** | **.000** | **.093** |  | **6.16** | **.004** | **.069** |
| Balloon x Drug | 2 | 1.05 | .343 | .013 |  | 1.37 | .256 | .016 |  | 0.24 | .748 | .003 |
| Balloon x Affect | 2 | .10 | .881 | .001 |  | 1.05 | .350 | .013 |  | 0.69 | .480 | .008 |
| Balloon x BMI | 2 | .44 | .614 | .005 |  | 0.11 | .894 | .001 |  | 1.81 | .173 | .021 |
| Balloon (Error) | 166 |  |  |  |  |  |  |  |  |  |  |  |

**Note**: *Drug* was coded 0=Placebo, 1=Propranolol. *Affect* refers to post-stressor mean negative, high arousal affect. *BMI* refers to body mass index. Given that *balloon type* was significant in Mauchly’s test of sphericity, model *p*-values reported use the Greenhouse-Geisser correction. Significant effects are bolded.

**Table S4.** Unadjusted (i.e., no covariates included) mixed effects ANOVAs assessing overall effects of propranolol, balloon type, and BELT task phase on BELT points earned, pumps made, and explosions, reported for future meta-analytic purposes.

|  |  | **Points Model** | | |  | **Pumps Model** | | |  | **Explosions Model** | | |
| --- | --- | --- | --- | --- | --- | --- | --- | --- | --- | --- | --- | --- |
| ***Predictors*** | ***df*** | ***F*** | ***p*** | ***partial η^2^*** |  | ***F*** | ***p*** | ***partial η^2^*** |  | ***F*** | ***p*** | ***partial η^2^*** |
| ***Between-subject effects*** |  |  |  |  |  |  |  |  |  |  |  |  |
| Intercept | 1 | **1818.90** | **.000** | **.955** |  | **1680.19** | **.000** | **.952** |  | **398.81** | **.000** | **.824** |
| Drug | 1 | **4.70** | **.033** | **.052** |  | 1.60 | .209 | .019 |  | 0.27 | .606 | .003 |
| Error | 85 |  |  |  |  |  |  |  |  |  |  |  |
|  |  |  |  |  |  |  |  |  |  |  |  |  |
| ***Within-subject effects*** |  |  |  |  |  |  |  |  |  |  |  |  |
| Balloon | 2 | **247.14** | **.000** | **.744** |  | **79.93** | **.000** | **.485** |  | **150.33** | **.000** | **.639** |
| Balloon x Drug | 2 | 2.47 | .106 | .028 |  | 2.12 | .144 | .024 |  | 0.28 | .688 | .003 |
| Balloon (Error) | 170 |  |  |  |  |  |  |  |  |  |  |  |
|  |  |  |  |  |  |  |  |  |  |  |  |  |
| Task phase | 2 | **29.99** | **.000** | **.261** |  | **8.16** | **.002** | **.088** |  | **10.15** | **.000** | **.107** |
| Task phase x Drug | 2 | 1.66 | .193 | .019 |  | 0.37 | .620 | .004 |  | 2.90 | .058 | .033 |
| Task phase (Error) | 170 |  |  |  |  |  |  |  |  |  |  |  |
|  |  |  |  |  |  |  |  |  |  |  |  |  |
| Balloon x Task | 4 | **10.67** | **.000** | **.112** |  | **27.14** | **.000** | **.242** |  | **10.30** | **.000** | **.108** |
| Balloon x Task x Drug | 4 | 1.39 | .240 | .016 |  | **3.58** | **.017** | **.040** |  | 1.61 | .182 | .019 |
| Balloon x Task (Error) | 340 |  |  |  |  |  |  |  |  |  |  |  |

**Note**: *Drug* was coded 0=Placebo, 1=Propranolol. *Balloon* included three types: certain-long, certain-short, and uncertain. *Task* included three phases or averaged timepoints: the early task phase, mid-task phase, and the last or final task phase. Given that the repeated measures of *balloon type* and *task phase* were significant in Mauchly’s test of sphericity (*ps*<.000), model p-values reported use the Greenhouse-Geisser correction. Significant effects are bolded.

**Table S5.** Unadjusted (i.e., no covariates included) univariate ANOVAs probing overall effects of propranolol on BELT pumps in the late task phase split by balloon type, reported for future meta-analytic purposes.

|  |  | **Long-Certain Balloon Model** | | | **Short-Certain Balloon Model** | | | **Uncertain Balloon Model** | | |
| --- | --- | --- | --- | --- | --- | --- | --- | --- | --- | --- |
| ***Predictors*** | ***df*** | ***F*** | ***p*** | ***η^2^*** | ***F*** | ***p*** | ***η^2^*** | ***F*** | ***p*** | ***η^2^*** |
| ***Between-subject effects*** |  |  |  |  |  |  |  |  |  |  |
| Intercept | 1 | **516.30** | **.000** | **.859** | **3758.81** | **.000** | **.978** | **1231.19** | **.000** | **.935** |
| Drug | 1 | 4.03 | .048 | .045 | 0.40 | .530 | .005 | 0.16 | .688 | .002 |
| Error | 85 |  |  |  |  |  |  |  |  |  |

**Note:** *Drug* was coded 0=Placebo, 1=Propranolol. Significant effects are bolded.
